# Clinical applications and utility of cell-free DNA-based liquid biopsy analyses in cervical cancer and its precursor lesions

**DOI:** 10.1038/s41416-022-01868-6

**Published:** 2022-06-20

**Authors:** Johanna Herbst, Klaus Pantel, Katharina Effenberger, Harriet Wikman

**Affiliations:** Department of Tumour Biology, University Medical Centre Hamburg-Eppendorf, Hamburg, Germany

**Keywords:** Oncology, Predictive markers

## Abstract

Human papilloma virus (HPV) is an infectious carcinogenic agent. Nearly all cervical cancers are positive for one of the high-risk HPV subtypes. Although the introduction of the HPV vaccines in many countries have shown tremendous positive effects on the incidence of both cervical intraepithelial lesions (CIN) and invasive cancer, the large majority of females worldwide are still not vaccinated. Patients with diagnosed high-grade CIN need a lifelong close monitoring of possible relapse or development of invasive cancer. Different blood-based liquid biopsy approaches have shown great promise as an easily obtainable minimally invasive tool for early detection and monitoring of disease. Among the different liquid biopsy approaches the clinical relevance of cell-free DNA (cfDNA) in cervical cancer has been best investigated. In cervical cancer, the DNA fragments can be of both, human as well as viral origin. Thus, the mutation and methylation status of genes related to carcinogenesis as well as the HPV status can be analysed in plasma from cervical cancer patients. This review describes recent advances in different cfDNA approaches for early detection and monitoring of cervical cancer and its precursor lesions.

## Introduction

Cervical cancer is the fourth most common female cancer worldwide, with 570,000 new cases annually, and the fourth leading cause of cancer-related deaths for women [1]. The average age of onset is 50 years and thus clearly lower compared to other common cancers [2]. Development of cervical cancer is closely linked not only to human papilloma virus (HPV) infection but also to the Human Development Index as most cases of cervical cancer occur in women in developing countries [3].

HPV is an infectious carcinogenic agent. It infects different epithelial sites and can cause cancer mainly in the cervix, anus or oropharynx [4]. Virtually, all biopsies of cervical cancers are found positive for HPV [5]. The group of HPVs contains more than 120 subtypes. Based on their cancer risk, they are divided into low and high risk (hrHPV). Today, 12 different HPV subtypes are classified oncogenic (HPV16, 18, 31, 33, 35, 39, 45, 51, 52, 56, 58 and 59) [6]. Of these hrHPV, HPV16 and HPV18 are especially prevalent and responsible for 74% of all cervical cancers [7]. Submitted sexually, hrHPV reaches the transformation zone of the cervix and enters the basal layer of the epithelium [8]. During the infection, upregulation and consequently overexpression of the viral oncogenes E6 and E7 lead to deactivation of tumour-suppressor genes TP53 and RB, which finally causes inhibition of cell cycle control and proliferation, immortalisation and inhibition of apoptosis [9].

The well-described precancerous lesions of cervical cancer can be classified into different grades. These cervical intraepithelial lesions (CIN) can evolve from low-grade (LSIL/CIN1) to high-grade squamous intraepithelial lesions (HSIL/CIN2/CIN3). They develop from HPV-positive cells over several months and transform in 30% of patients with a persistent HPV infection into an invasive cervical cancer [10]. The introduction of the 2-valent HPV vaccine in 2006/2007 has had a huge impact on HPV infection rates and cervical cancer occurrence [11, 12]. Still notably, the currently recommended 9-valent vaccination does not cover all important hrHPV subtypes, and a large part of the population has not received the vaccination and thus has a risk of developing CIN and cancer [13].

Different liquid biopsy (LB) approaches have recently shown great promise as an easily obtainable minimally invasive tool for early detection and disease monitoring [14]. In plasma, cell-free DNA (cfDNA) circulates as short DNA fragments originating from very different origins, including viral DNA. Also, tumours spread DNA fragments into the blood (circulating tumor DNA (ctDNA)), which can be specifically detected and analysed [15].

In the field of liquid-based tests in cervical neoplasia, it is important to distinguish between liquid-based cytology (LBC) and LB on blood samples. LBCs are smear tests from the cervical uterine stored in liquid medium and usable not only for cytology screening but also for PCR-based DNA and methylation analysis [16–19]. In this review, we discuss recent advances in the use of mainly blood-based cfDNA LB approaches for early detection and monitoring of CIN and invasive cervical cancer.

## Principles and clinical utility of blood-based LB analyses in cancer

**Fig. 1:**
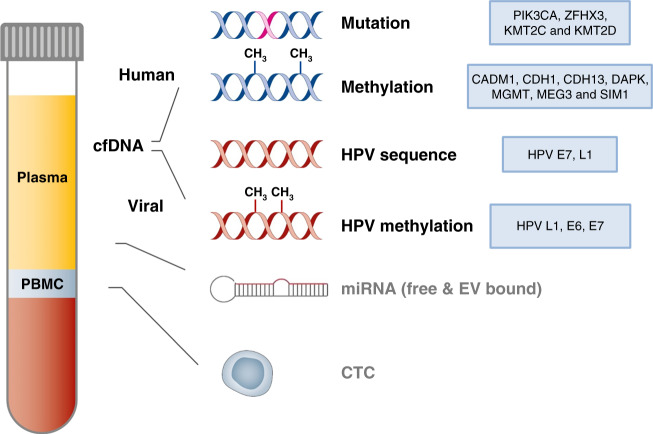
Contents of the liquid biopsy from blood. Blood-based liquid biopsy refers to the detection of cfDNA, disseminated and circulating tumour cells (CTCs), miRNAs and extracellular vesicles (EV). Within the fraction of cfDNA, markers of human as well as of viral origin can be found in cervical neoplasias. In cervical cancer, analyses on cancer-related mutations (e.g., PIK3CA, ZFHX3, KMT2C, KMT2D), methylation (e.g., CADM1, CDH1, CDH13, DAPK, MGMT, MEG3, SIM1), different HPV subtypes (HPV E7, L1) and methylated HPV (HPV L1, E6, E7) have been analysed in patient blood samples. Recent advances in CTCs and miRNA analyses in cervical neoplasias have recently been reviewed by [27]. PBMC peripheral blood mononuclear cells, cfDNA cell-free DNA, miRNA microRNA.

The term *liquid biopsy* (LB) was introduced already 10 years ago [20] and refers to the detection of cancer-related biomolecules/cells/cell parts such as cfDNA, disseminated and circulating tumour cells (CTCs), miRNAs and extracellular vesicles in blood and other body fluids (Fig. 1). Since then, increasing interest in this field has taken place due to the great potential in early cancer detection, disease prognosis, monitoring response and resistance to treatment or detecting minimal residual disease [21].

The large majority of studies published today still deals with mutation detection [22]. Additional features that can be analysed in cfDNA are chromosomal aberrations, epigenetic changes like methylation patterns and DNA fragment lengths, as well as next-generation sequencing (NGS) depth coverage giving information about tumour-specific gene expression [15]. In viral-related cancers, the non-human origin of the viral DNA enables the use of HPV-DNA as a specific biomarker in LB-based assays [23]. Plasma Epstein–Barr virus DNA analyses have been successfully implemented in large cohort screenings for early detection of nasopharyngeal carcinomas [24, 25].

Recent data have shown that ctDNA mutation analysis for screening can be hampered by the notion of the commonly occurring clonal haematopoiesis resulting in mutations in known tumour suppressor and oncogenes in normal cells. Therefore, methylation-based LB approaches gain increasing popularity for early detection and screening [23]. Recently, targeted sequencing of >100,000 methylation targets in cfDNA of 6689 participants was performed, and >50 types of cancer could be detected across all stages at a high specificity of >99% and sensitivity of 43.9–54.9% [26]. Similarly, as the detection of viral DNA in blood or saliva might be another powerful and much cheaper monitoring option in some cancer types. Based on these reports and, the feasibility of population-wide screening using LB for adding important clinical information to diagnosis, prognosis and early disease screening, the way from bench to bedside is paved. Blood withdrawal tests can easily be integrated into routine physicians’ exams.

At this point, a sentence of caution should be made: the studies in the field of LB in cervical neoplasias have often been carried out with a limited number of patients [27] for mainly two reasons: First, cervical cancer has become rarer in developed countries, hence getting samples becomes more time consuming; and second, the different cfDNA-related technologies in blood and other body fluids from patients especially with precancerous lesions is still in development.

## HPV-ctDNA detection in blood of CIN and cervical cancer patients

Owing to the non-human origin of HPV-DNA, small panels or even single site assays can be utilised in LB approaches—a great advantage for cost effectiveness and sensitivity. Therefore, most published studies in cervical cancer patients have used different PCR-based methods including quantitative PCR or droplet digital PCR (ddPCR)-based methods, which can reach a sensitivity of <0.01% [15].

Most of these studies though have been rather small, with only 5 studies analysing >100 patients (reviewed in [23, 28]). The majority has used primers detecting regions in the L1, E6 or E7 genes in HPV16 and HPV18. The positivity rates ranged between 11 and 90%. This large variation could be accounted for by mainly technical aspects but also the number of different hrHPV subtypes analysed.

Hsu et al. collected blood samples before surgery from 112 stage IB or IIA patients and 20 CIN patients [29]. None of the CIN patients and only 24.1% of carcinoma patients were positive for HPV-ctDNA. Still, serum HPV-ctDNA correlated with poor prognosis factors that warrant adjuvant therapy. A lower sensitivity may be explainable using only 200 μL serum. Dong et al. analysed the incidence of HPV16 and HPV18 E7-ctDNA in plasma of 175 carcinoma and 57 CIN patients by conventional PCR [30]. Again only 6.9% of the carcinomas and 1 CIN patient were found positive.

Cheung et al. analysed blood collected before treatment from 138 cervical cancer patients (stages I–V) for HPV E7 and L1 sequences [31]. With 61.6%, a much higher sensitivity for HPV-ctDNA was reached using ddPCR. Patients with high viral load showed an increased risk of recurrence and death at 5 years in univariate but not in multivariate analysis.

In a recent study, Cabel et al. showed that HPV-ctDNA can be detected before chemo-radiotherapy in 69% patients diagnosed with locally advanced cervical cancer [32]. HPV-ctDNA level was correlated significantly with HPV copy number in the tumour, with lowest levels found among HPV18-positive patients. Furthermore, residual HPV-ctDNA levels after treatment and during follow-up had a prognostic impact. Similar results were published by [33], both indicating that most patients have a clearance of HPV-ctDNA at the end of treatment and those with persistent HPV ctDNA at treatment end or during FUP may help to classify patients with high relapse risk.

Interestingly, Cocuzza et al. analysed plasma from 120 women diagnosed with low-grade or precancerous cervical lesions [34]. The authors used a real-time quantitative TaqMan PCR assay detecting seven different HPV subtypes. In 41 patients (34.2%), HPV-ctDNA could be detected and quantified in plasma samples. These results indicate a potential use of LB analyses for pre-screening in parallel with cervical smears.

A recent meta-analysis combined the data from 10 different studies comprising data from 684 cervical cancer patients [28]. Pooled sensitivity and specificity were 0.27 (95% confidence interval (CI), 0.24–0.30) and 0.94 (95% CI, 0.92–0.96), respectively. In the included studies, HPV-ctDNA showed not only clear diagnostic value for diagnosing and monitoring cervical cancer but also the need of further optimisation of LB analyses to achieve a higher sensitivity. The data on HPV-ctDNA was comprised in a review describing detection techniques (classic/reverse transcriptase–PCR and newer ddPCR/NGS) and outcomes in studies among 16–138 cervical cancer patients. The authors conclude that ddPCR and NGS have made it possible to detect HPV subtypes and their integration status by plasma sampling and consequently may impact clinical decision making [35]. Another review/meta-analysis focussed on HPV16/HPV-ctDNA in blood as well and stated that its occurrence was significantly correlated to HPV-associated cancer compared to healthy donors [36].

Taken together, detection of viral DNA in plasma and in certain cases saliva in viral-related cancer has shown high specificity and even potential for early screening. Furthermore, in terms of the clinical utility, presence of HPV-ctDNA seems to be clearly associated with poor outcome in cervical neoplasias. LB facilitates easy consecutive analyses by which monitoring of HPV-ctDNA may be used as a marker of therapy response or failure, and it may be used as an indicator of persistent residual disease.

## Analyses of methylation in blood of CIN and cervical carcinomas

Epigenetic alterations such as methylation and histone acetylation are important mechanisms of gene regulation and thus play a crucial role also in carcinogenesis [37]. Both the host and HPV genomes are epigenetically modified during an HPV infection [38]. E6 and E7 both can modify the DNA methylation patterns of infected cells. E7 can directly bind to and activate *DNA methyltransferase 1* (*DNMT1*), resulting in methylation of multiple genomic host sites. *DNMT1* is the major enzyme responsible for maintaining methylation patterns following DNA replication by mediating the transfer of a methyl group to cytosines. Also, E6 can, via suppression of p53, induce the expression of *DNMT1*. E7 can furthermore induce the expression of KDM6A or 6B causing a histone demethylation of certain target genes, such as p16. The resulting p16 overexpression is widely used as a surrogate for hrHPV infection and transformation, together with the proliferation marker Ki67. In general, for cervical cancer screening of tissues and LBC, an increased methylation of both host and viral sequences has been associated with increased invasiveness [38–41].

In contrast to several LB methylation analyses of human genes, to our knowledge only one pilot study assessed the HPV methylation status. Paired LBC, serum and urine samples from CIN patients were tested for HPV16-L1 gene methylation in six samples. Here HPV16-L1 methylation could discriminate normal cytology samples from premalignant cervical lesions with high sensitivity and specificity [42].

Hypermethylation of CpG islands in promoter regions of several human genes has been found during large genome-wide studies of methylation profiles and a correlation to cervical cancer or precancerous CIN lesions has been described [43–45]. The most frequently tested host-genome methylation markers in cervical smears and tumours are *cell adhesion molecule 1* (*CADM1*) and *T-lymphocyte maturation-associated protein* (*MAL*). Overmeer et al. tested a bi-marker panel for *CADM1* and *MAL* in a population-based screening [46]. They found that in tissue the CADM1/MAL combination was superior to discriminate each, CIN3 and cervical cancer from normal tissue/CIN1 with methylation-positivity rates of 97% (CIN3) and 99% (cervical cancer). Other genes that have shown prognostic relevance and significance in smears include *CADM1*, *MAL*, *FAM19A4*, *EPB41L3*, *JAM3*, *DAPK*, *PAX1* and *CDH1* [38, 46–48].

To enable sufficient accuracy often also gene panels have been used [49]. For example, the GynTect® assay detecting methylation in a 6-marker-panel (*ASTN1*, *DLX1*, *ITGA4*, *RXFP3*, *SOX17* and *ZNF671*) was compared to CINtec Plus® (tissue diagnostic for Ki-67/p16) and cobas® HPV (PCR-based hrHPV-subtype analysis) in terms of specificity to detect cancer or CIN in smears [50]. GynTect® was the most powerful method with a specificity of 94.6% in detecting CIN3 (compared with 69.9% for CINtec Plus® and 82.6% for cobas® HPV, respectively). Interestingly, a recent meta-analysis showed that detecting CIN3+ via DNA methylation shows promising results with higher sensitivity than testing for the HPV16 and HPV18 genotypes and beats cytology testing in the range of specificity [51] thus making analysis of methylation markers also in LB highly interesting.

In Table 1, we have listed studies, which have analysed host-genome methylation in LB of CIN and cervical cancer [42, 52–57] (Table 1). *CADM1* promoter hypermethylation was analysed in plasma by [57] using quantitative methylation-specific PCR. The authors collected peripheral blood of CIN1, CIN2/CIN3, cervical cancer and healthy women before any kind of treatment. Plasma CADM1 methylation levels were significantly higher in cancer patients compared to benign disease groups (*p* < 0.001) and levels were significantly higher in patients with lymph node or distant metastases (*p* = 0.0049 and *p* < 0.001, respectively). When measured in combination with plasma D-dimer levels, it reached a sensitivity of 80.4% and specificity of 90.5% for metastasis prediction in cervical cancer patients [57].

**Table 1:** Liquid biopsy-analysed genes for host genome methylation in cervical cancer and CIN.

Ref.	Gene	Sample type	CC (*n*)	CIN (*n*)	HD (*n*)	Detection method	Positivity rate	Concordance to tissue	Clinical association
[57]	CADM1	Plasma, tissue	190	53 (CIN1), 49 (CIN2/3)	70	qMSP	N/A	93.3% (*n* = 45)	Sign. associated with increasing malignancy. 53.6% sensitivity and 91.4% specificity to predict metastasis. Combined CADM1 methylation and D-dimer-level analysis: 80.4% sensitivity and 90.5% specificity
[52]	CDH1, CDH13	Serum	93	0	0	Real-time PCR	CDH1: 42%; CDH13: 4%	N/A	CDH1/CDH13 methylation status is significantly associated with disease-free survival
[53]	DAPK	Plasma, tissue	40	0	30	MSP	40.0%	64.3% (*n* = 23)	Combined methylation of DAPK, p16 and MGMT showed concordance to tissue in 55%. HD plasma was not methylated
	p16	Plasma, tissue	40	0	30	MSP	10.0%	33.3% (*n* = 23)	
	MGMT	Plasma, tissue	40	0	30	MSP	7.5%	25% (*n* = 23)	
[54]	MEG3	Plasma, tissue	168	84 (CIN1/2) 76 (CIN3)	168	MSP	90.5% (CC); 89.5% (CIN3), 35.7% (HD)	Sign. correlation (*p* < 0.001, *R* = 909)	MEG3 methylation discriminates CIN3 from HD and is associated with worse overall survival in CC patients
[56]	SIM1	Plasma, tissue	41	0	0	qMSP	36.60%	41.5% (*n* = 41)	SIM1 metyhlation detection showed a 38.5% sensitivity and 100% specificity. Leukocytes from patients did not show methylation
[42]	ZNF516, FKBP6, INTS1, HPV16-L1	Plasma, urine	40 (total)			qMSP	85.7%	N/A	Panel obtained a 85.7% sensitivity and 60.9% specificity (AUC of 0.807, PPV of 40% and an NPV of 93.3%) compared to women with no lesion/CIN1 to CIN3/CC

*CC* cervical carcinoma, *CIN* cervical intraepithelial lesion, *HD* healthy donor, *MSP* methylation-specific PCR, *N/A* not available, *Ref.* reference.

Another marker frequently tested in tissue and cervical scrapings is methylation of *death-associated protein kinase* (*DAPK*) reviewed by [39]. In 2004, Yang et al. compared tumour tissue and plasma and found that *DAPK* methylation was detectable in 60% of tissue DNA and in 40% of plasma DNA from 40 carcinoma patients [58]. A 63% concordance between plasma and tissue was observed. Healthy donor plasma was also analysed and showed no methylation at all.

Promoter methylation of the *E-cadherin gene* (*CDH1*) was tested in combination with *cadherin 13* (*CDH13*) in serum samples of 93 cervical cancer patients [52]. Hypermethylation was found in 42% (*CDH1*) and 4% (*CDH13*), respectively. The study could show a better clinical outcome of cervical cancer patients with unmethylated *CDH1/13* compared to patients with the methylated genes (median disease-free survival for *CDH1/13* unmethylated: 4.3 years, methylated: 1.2 years).

*Single-minded homolog 1* (SIM1) was mentioned in only two studies so far for cervical cancer tissue [56, 59]. Kim et al. also analysed plasma samples and detected in 36.6% of the samples SIM1 methylation with high specificity (100%) but low sensitivity (38.5%) [56].

In a large study, the methylation level of m*aternally expressed 3* (*MEG3)* in plasma from 168 cervical cancer patients correlated with the diagnosis of cervical cancer (area under the curve (AUC) 0.867) [54]. In healthy donors and 84 CIN1-2, methylation levels were low and with no significant differences but significantly increased in CIN3 (*n* = 76; *p* < 0.001). Plasma *MEG3* hypermethylation was a risk factor for CIN3, hrHPV-infection and lymph node metastasis (AUCs 0.788, 0.730 and 0.804, respectively) and associated with a poorer recurrence-free (*p* = 0.0004) and overall survival (OS; *p* = 0.0013).

Guerrero-Preston et al. tested a three-marker panel with z*inc finger protein 516* (*ZNF516*)*, FKBP prolyl isomerase family member 6* (*FKBP6*), and *integrator complex subunit 1* (*INTS1*) together with HPV16 L1 region for urine cfDNA and plasma cfDNA to prove their previous results from smear and tissue samples [42]. This panel showed 85.7% sensitivity, 60.9% specificity and an AUC of 0.807 for the detection of CIN2+ lesions in plasma samples. In urine cfDNA, the results were slightly better with 75% sensitivity, 83.3% specificity and an AUC of 0.86.

Besides blood, hrHPV subtypes as well as DNA hypermethylation for a 6-marker-panel, i.e., *FAM19A4*, *GHSR*, *PHACTR3*, *PRDM14*, *SST*, and *ZIC1*, were tested in the urine of 41 cervical cancer patients. A strong correlation was found for hrHPV in urine and corresponding cervical scrapings (kappa = 0.79). Detection of methylation in urine also correlated moderately or strongly to findings in scrapes (*r* = 0.508–0.717), and the methylation panel was capable of discriminating cancer from normal control samples (AUC = 0.744–0.887) [60].

In summary, as methylation is an early and specific epigenetic event in cervical carcinogenesis, methylation-based analysis could serve as valuable clinical tool for early disease detection and diagnosis. However, the analysis of methylation status in LB samples from cancer patients is still rather challenging, with room for technical improvement [23]. We identified six studies analysing methylation of either single genes or a set of genes in human blood of patients with cervical neoplasias. In total, 11 different genes have been assessed with respect to their methylation status in cfDNA. Although most studies used small numbers of retrospectively collected samples, these studies already showed promising perspectives for detecting or monitoring cervical lesions. Further evaluation in larger studies is still needed to proof the detectability and specificity, e.g. in comparison to mutations of HPV-ctDNA.

## ctDNA mutation analyses in LB of CIN and cervical cancer

Integration of the HPV genome into cervical cells typically results in an increased expression and stability of transcripts encoding the viral oncogenes E6 and E7. This integration preferentially favours common fragile sites [61] and is known to induce DNA damage, centrosome abnormalities and chromosomal mis-segregation causing chromosomal instability. Therefore, not surprisingly, cervical cancers show a high degree of chromosomal alterations and an APOBEC cytidine deaminase mutagenesis pattern [62–64]. Indeed, in the integrative molecular analyses of the complete set of tumours in The Cancer Genome Atlas, cervical squamous tumours clustered in high aneuploidy clusters, which are defined by high proliferation and DNA repair pathway alterations and basal signalling [65].

In general, most tumours have their own mutation profiles, and few genes are commonly found in all tumours although, like most other carcinomas, ERBB2/PI3K/AKT/mTOR are most affected. Three larger whole-exome sequencing-based tumour profiling efforts of cervical tumours have been published [63, 66, 67]. Although surprisingly large differences in mutation frequencies for the different target genes were reported, all studied identified hotspot (recurrent) mutations in >10% of the samples only in the *serine/threonine protein kinase PIK3CA* gene.

This fact makes LB-based single gene mutation detection approaches challenging. Chung et al. explored the feasibility of *PIK3CA* mutation testing by ddPCR in cervical cancer patients. Two *PIK3CA* mutations, p.E542K and p.E545K, were measured in cfDNA in pre-treatment plasma of 177 patients with primary invasive cervical cancer. Mutations were detected in 22.2% of the samples and correlated to median tumour size and decreased disease-free survival [68].

Seven other studies using NGS approaches have been recently published [69–75]. Lee et al. designed an NGS panel of 24 genes associated with cervical cancer. In 18/24 patients, mutations could be detected [71]. The most frequent mutations were *ZFHX3*, *KMT2C* and *KMT2D*, all found in >75% of the samples. Tian et al. used a targeted exome-sequencing approach analysing 48 tumour relevant genes [74]. They developed an algorithm to assess the cumulative mutation fraction of all covered positions (>150,000 bp/sample) and the deviation of tumour mutation fraction from the normal pattern (allele fraction deviation, AFD). Ninety-three plasma samples from 57 cervical cancer patients were analysed. This approach could monitor patient response to treatment and prognosticate tumour progression. Often, a low allele frequency deviation value at diagnosis followed by a later increase could successfully predict relapse [74].

In another study, the authors used a deep sequencing approach (CAncer Personalised Profiling by deep Sequencing (CAPP-Seq)) targeting 322 cancer-related genes in plasma samples from 82 locally advanced or metastatic cervical cancer patients. Mutations in five genes (*PIK3CA*, *BRAF*, *GNA11*, *FBXW7* and *CDH1*) correlated with a significantly shorter progression-free survival (PFS; *p* = 0.005) and OS (*p* = 0.007) in the metastatic patient cohort. Importantly, the authors show that longitudinal monitoring with ctDNA in LB samples can provide both predictive and prognostic information during treatment [75]. A CAPP-Seq-based NGS approach (197 gene panel) was also used in a small study [69] including four cervical cancer patients. All four patients showed non-synonymous mutations, but the number or type was not described.

Charo et al. described the ctDNA results from 105 gynaecologic cancer patients, including 13 cervical cancers (PREDICT trial NCT02478931). Two different panels consisting of either 54 or 73 genes were used. The most commonly found mutations among cervical cancer patients were *PIK3CA* (*n* = 8), *TP53* (*n* = 5), *FBXW7* (*n* = 3), *ERBB2* and *PTEN* (both *n* = 2). The concordance rate between tissue and plasma results in the whole study cohort ranged between 75 and 88%. Furthermore, higher mutant allele frequency was a significant independent prognostic factor for OS [hazard ratio (HR): 1.91, *p* = 0.03) [73].

In a recent very large pan-cancer ctDNA study, 123 cervical cancer plasma samples were included [72]. A deep sequencing approach analysing 1021 genes was used. Interestingly, variants related to clonal haematopoiesis (CH) were detected in 19% of cervical samples. CH-related mutations in *DNA Methyltransferase 3 Alpha* (*DNMT3A*) were most frequent in cervical cancers (5.9%). In general, cervical cancer had the fourth highest tumour mutational burden. The sensitivity of ctDNA mutation detection in M0 patients was 60.9% and >70% in metastatic patients.

Another group analysed preoperative plasma from 100 women with gynaecological cancers including 11 with cervical cancer for copy number alterations (CNAs). In 3/11 stage I–II patients, plasma CNAs could be detected. All patients with CNA had a shorter PFS and OS compared with those patients without CNA [70].

In conclusion, due to the large heterogeneity in mutation patterns in cervical neoplasias single-gene ctDNA approaches are not usually feasible. However, by using deep sequencing approaches analysing rather large gene panels one can obtain rather high sensitivities also in cervical cancer patients. Furthermore, these mutations are indicative of worse disease outcome in terms of progression as well as survival. However, such approaches are expensive and require corrections for possible CH, which increases even more the costs. Therefore, in cervical neoplasias, for routine clinical approaches a multi-analyte approach is most likely more sensitive.

## Conclusions and Outlook

Offering HPV vaccines and cervical screenings as a routine in gynaecologic surveillance has improved incidence and mortality of cervical cancer [12]. Yet, millions of females are diagnosed with high-grade CIN lesions each year [76]. Current routine screening consists of a combined approach to reveal cellular (cytology, Pap-test) and molecular (HPV-DNA) abnormalities in precancerous stages [77]. However, recent data have shown that sensitivity and specificity can even be increased by adding information on the methylation status [51], which, however, is not part of routine screening yet and may need further validation. As frequent smear taking is inconvenient, obtaining all relevant information by LB, here a simple blood draw, would be favourable. This could be included into the check-up by the physician.

In blood or other body fluids, DNA fragment shed from dysplastic or tumourigenous cells of human origin can be tested for mutations or methylation status. Viral DNA can be analysed for occurrence of oncogenic HPV subtypes and viral gene methylation. All these parameters enable a wide diagnostic and prognostic frame for the clinical management of cervical neoplasias. The ctDNA fraction of cfDNA varies between cancer types as well as between patients with the same cancer type, but in general the amount increases with more advanced disease stages [15]. Thus, for detection of early stages such as CIN lesions, highly sensitive and specific assays are obviously needed.

Most LB-related data have been collected for the use of HPV-DNA as a detection marker in the blood of cervical cancer patients. Many studies, including two recent meta-analyses [28, 36], have shown that not only HPV-ctDNA assays provide a diagnostic value for diagnosing and monitoring cervical cancer but also that there is a need of further optimisation of the analyses to achieve higher sensitivity. Especially in case of CIN, a low sensitivity is yet problematic. More sensitive approaches like ddPCR or ultra-deep sequencing could overcome this issue. The feasibility of detecting viral DNA in plasma for screening approaches has been shown for nasopharyngeal carcinoma (NPC) [25]. Thus, large prospective studies such as those performed on high-risk NPC populations need to be more frequently performed, e.g. in CIN3 patients, to evaluate the clinical relevance of the use of liquid biomarkers.

Methylation-based LB approaches, especially for early detection and screening, have recently gained an increasing interest due to the commonly occurring clonal haematopoiesis that can hamper mutation detection in ctDNA [15]. In cervical cancers, specific methylation changes of both host and viral genomes are well described [38, 44]. In the focus of viral methylation detection are the E2-binding-site-1 and the late viral regions L1 and L2 of different hrHPV subtypes [41]. However, LB approaches showing clinical utility are still missing. So far, markers for methylated human genes with prognostic relevance in blood-based testing include C*ADM1*, *DAPK* and *CDH1*. Especially for *CADM1*, multiple studies have been published showing that this marker can differentiate between benign and malignant cervical disease and may function as a tumour metastasis marker in blood [57]. Still a panel of selected genes could be the most promising LB approach. In tissue and smear, different panels have been tested reaching a sensitivity and specificity close to 100% [50]. A hybrid panel including human and viral genes might be more favourable to improve sensitivity and specificity.

Cervical tumours are characterised by a high degree of heterogeneity in mutation patterns between different patients [63] with only a few recurrent mutations found, making LB-based mutation detection approaches very challenging. In ctDNA, *PIK3CA*, *ZFHX3*, *KMT2C* and *KMT2D* were revealed as the most frequent mutations yet not affecting all patients [67]. Taken together, an optimal ctDNA mutation panel and platform for cervical neoplasia screening still needs to be developed.

In summary, the range of current LB markers in cervical neoplasias encompasses both host and viral genome analyses. Further studies must show which of these (combined) approaches can build a strategy to improve screening or diagnosis and will have clinical relevance in the management of cervical disease as new biomarkers. A promising future strategy might be the combination of cervical smears and blood analyses for initial diagnosis especially of CIN and a combined multi-disciplinary molecular LB-based platform for monitoring representing a “cervical disease management 2.0”. A simple blood draw would not only relieve the patients but also the health care system. Such a new platform may even have the potential of being used as a triage tool for CIN bearing a high or low risk for CC. Still, optimised assays, larger well-designed retrospective and prospective studies need to be performed to prove the ultimate clinical utility of the different (combined) LB approaches.

## Data Availability

Not applicable.
